# Biodegradation and decolourization of textile dye wastewater using *Ganoderma lucidum*

**DOI:** 10.1007/s13205-012-0073-5

**Published:** 2012-06-13

**Authors:** Sathian Selvakumar, Rajasimman Manivasagan, Karthikeyan Chinnappan

**Affiliations:** Environmental Engineering Laboratory, Department of Chemical Engineering, Annamalai University, Annamalai Nagar, 608 002 Tamil Nadu India

**Keywords:** Optimization, RSM, *Ganoderma lucidum*, Dye wastewater, COD

## Abstract

In this work, treatment of textile dye wastewater was carried in a batch reactor using *Ganoderma lucidum*. The characteristics of textile dye wastewater were studied. The effect of process parameters like pH, temperature, agitation speed and dye wastewater concentration on dye decolourization and degradation were studied. These parameters were optimized using response surface methodology (RSM). From the results, the optimized conditions were: pH 6.6, temperature 26.5 °C, agitation speed 200 rpm and dye wastewater concentration 1:2. At these optimized conditions, the maximum decolourization and COD reduction were found to be 81.4 and 90.3 %. Kinetic studies were carried out using different models like first-order, diffusional and Singh model. From the results, it was found that the degradation follows the first-order reaction model.

## Introduction

Synthetic dyes are widely used in several industries such as textile, paper, printing, cosmetics, pharmaceuticals, colour photography and petroleum (Marmion 1991). Dyes are classified into acidic, basic, disperse, azo, diazo, anthraquinone and metal complex based on their structure. On the basis of the dyeing process, textile dyes are classified as reactive, direct, disperse, acid, basic and vat dyes (Campos et al. 2001). Textile industries utilize large amounts of water during processing and also generate substantial amounts of wastewater (Hutton 1972). About 10–15 % of the dyes are lost in the wastewater during the dyeing process (Zollinger 1987). Coloured wastewater from the textile industries is one of the most obvious indicators of water pollution. Coloured dye wastewater causes severe effects on aquatic environment even in small amounts. Apart from the colour, the dischargeable dye wastewater also contains other pollutants like degradable organics, nutrients, pH altering agent, salts, sulphur, toxicants and refractory organics (Somasiri et al. 2008; Haroun and Idris 2009).

In general, physical, chemical and biological methods are used to treat the textile industry wastewater. Physical and chemical methods include adsorption, chemical precipitation, flocculation, photolysis, chemical oxidation and reduction, electro-chemical treatment and ion-pair extraction (Azmi et al. 1998; Moreria et al. 2000; Rajeshkannan et al. 2010, 2011). These methods are mostly ineffective, expensive, produce side reactions, high sludge and by-products, not suited to degrade all dyes, etc. (Krull et al. 1998; Verma and Madamwar 2003). Hence, researchers have focused on biological treatment as the best alternative. The operational cost is relatively low when compared with conventional technologies (Arutchelvan et al. 2003; Jadhav and Govindwar 2006). Many microorganisms, including bacteria, fungi and actinomycetes, have been reported for their ability to decolourize dyes (Chang et al. 2001; Khehra et al. 2005). Among these microorganisms, white rot fungi are the most intensively studied dye decolourizing microbes. These fungi produce large quantities of extracellular enzymes that help to remove dyes from industrial effluent and also have the ability to resist unfavourable environmental conditions (Pointing 2001; D’Souza et al. 2006).

In this study, a white rot fungal strain, *Ganoderma lucidum*, was examined for its ability to decolourize the textile dye in industry wastewater. The effect of process variables on textile dye industry wastewater degradation was studied and optimized using response surface methodology (RSM).

## Materials and methods

### Textile dye wastewater

The textile dye wastewater was collected from a private small-scale industry located at Erode, Tamilnadu, India. The wastewater was analysed for various parameters as per the procedure given in APHA (1999), as given in Table 1. The wastewater was stored at 4 ± 1 °C in airtight plastic containers.

**Table 1:** Characteristics of textile dye industry wastewater

Parameters^a^	Values
pH	7.7–7.9
Colour	Brown
Total suspended solids	510
Total dissolved solids	3,880
BOD	1,132
COD	2,450
Sulphates	232
Chlorides	1,465

^a^All values except pH and colour are in mg/L

*Ganoderma lucidum* (MTCC- 1039) is a stock of the Microbial Type Culture Collection Centre (MTCC), Chandigarh, India. It is well preserved in the laboratory. The strain is maintained on solid medium at 4 °C. The media composition and process conditions were: agar 20 g/l; malt extract 20 g/l; temperature 25 °C; pH 6.5; incubation time 10 days.

### Response surface methodology (RSM)

The RSM has several classes of designs, with its own properties and characteristics. Central composite design (CCD), Box–Behnken design and three-level factorial design are the most popular designs applied by the researchers. The Box–Behnken design was used to study the effects of the variables towards their responses and subsequently in the optimization studies. This method is suitable for fitting a quadratic surface and helps to optimize the effective parameters with a minimum number of experiments, as well as to analyse the interaction between the parameters. In order to determine the existence of a relationship between the factors and the response variables, the data collected were analysed in a statistical manner using regression. A regression design is normally employed to model a response as a mathematical function (either known or empirical) of a few continuous factors and good model parameter estimates are desired.

The coded values of the process parameters are determined by the following equation

1 $$ x_{i} = \frac{{X_{i} - X_{0} }}{\Updelta X} $$

where *x*_*i*_ is the coded value of the *i*th variable, *X*_*i*_ the uncoded value of the *i*th test variable and *X*_0_ the uncoded value of the *i*th test variable at the centre point.

Regression analysis was performed to estimate the response function as a second-order polynomial

2 $$ Y \, = \, \beta_{0} \, + \, \sum\limits_{i = 1}^{k} {\beta {}_{i} } X_{i} + \sum\limits_{i = 1}^{k} {\beta_{ii} \, X_{i}^{2} } \, + \, \sum\limits_{i = 1,i \, < j}^{k - 1} {} \sum\limits_{j = 2}^{k} {\beta_{ij} \, X_{i} \, X_{j}} $$

where *Y* is the predicted response and *β*_*i*_, *β*_*j*_, *β*_*ij*_ are the coefficients estimated from regression. They represent the linear, quadratic and cross products of *x*_*1*_, *x*_*2*_, *x*_*3*_ on response.

The regression and graphical analysis with statistical significance were carried out using Design-Expert software (version 7.1.5, Stat-Ease, Inc., Minneapolis, USA). To visualize the relationship between the experimental variables and responses, the response surface and contour plots were generated from the models. The optimum values of the process variables were obtained from the response surface.

The adequacy of the models was further justified through analysis of variance (ANOVA). Lack-of-fit is a special diagnostic test for adequacy of a model that compares the pure error, based on the replicate measurements to the other lack-of-fit and model performance. *F* value, calculated as the ratio between the lack-of-fit mean square and the pure error mean square, is the statistic parameter used to determine whether the lack-of-fit is significant or not, at a significance level.

### Experimental procedure

Experiments were carried out in a 500-ml Erlenmeyer flask, according to the Box–Behnken design given in Table 2. The process parameters chosen for this study were pH, temperature, agitation speed and initial dye wastewater concentration. The pH of the sample was adjusted to 5, 7 and 9 by adding acid or base as required. Batch studies were carried out by varying the temperature to 23, 28 and 33 °C, respectively. Agitation speed was varied to 100, 200 and 300 rpm by means of an agitator. The initial concentration of dye wastewater was varied as 1:1 and 1:2. The dye concentration was measured with Bio-Spectrophotometer (Model: BL-200, ELICO, India) at a wavelength of 395 nm. COD of the sample was analysed using the procedure given in APHA.

**Table 2:** Box–Behnken design-based experimental conditions and results for textile dye wastewater treatment using *Ganoderma lucidum*

Run no.	*A*-pH	*B*-Temperature	*C*-Agitation speed	*D*-Dye wastewater concentration	% decolourization		% degradation	
					Experimental	Predicted	Experimental	Predicted
1	5	28	150	1:2	75.3	71.49	81.4	81.06
2	9	23	150	1:1	60.3	57.25	68.5	66.47
3	5	28	150	Raw	40.2	38.12	51.8	47.11
4	7	33	150	Raw	45.6	48.91	58.8	58.32
5	7	28	200	Raw	49.6	50.29	60.2	62.65
6	9	28	100	1:1	56.3	60.33	63.8	64.34
7	7	28	100	1:2	78.5	74.81	84.5	84.28
8	7	23	150	1:2	79.5	81.11	88.9	88.44
9	7	23	150	Raw	40.2	39.44	57.5	58.49
10	7	23	200	1:1	65.3	66.89	74.2	75.95
11	9	28	200	1:1	62.3	61.93	69.5	67.30
12	7	28	150	1:1	70.3	70.07	78.8	78.80
13	7	28	150	1:1	69.6	70.07	78.9	78.80
14	7	33	100	1:1	62.7	59.20	70.5	67.46
15	9	28	150	1:2	66.8	66.97	72.5	75.90
16	7	33	150	1:2	59.8	65.48	87.5	85.57
17	9	33	150	1:1	60.2	57.52	65.2	66.45
18	9	28	150	Raw	40.2	42.10	53.6	52.66
19	7	28	150	1:1	70.3	70.07	78.7	78.80
20	5	23	150	1:1	61.2	60.87	66.8	67.78
21	7	33	200	1:1	71.2	68.35	79.6	79.53
22	7	28	200	1:2	79.2	79.25	88.9	88.45
23	5	28	200	1:1	64.3	65.19	72.6	71.12
24	7	23	100	1:1	65.9	66.84	75.3	74.08
25	7	28	100	Raw	48.6	45.54	50.2	52.88
26	5	28	100	1:1	52.3	57.59	58.9	60.15
27	5	33	150	1:1	54.4	54.44	60.5	64.77
28	7	28	150	1:1	70.1	70.07	78.4	78.80
29	7	28	150	1:1	70.5	70.07	78.8	78.80
30	7	28	150	1:1	70.0	70.07	78.0	78.80

### Laccase enzyme assay

The laccase activity was determined using 2, 2′-azino-bis (3-ethylbenzthiazoline-6-sulphonic acid) (ABTS) as the substrate (Rasera et al. 2009). The laccase reaction mixture contained 0.5 ml of 0.45 mM ABTS, 1.2 ml of 0.1 M phosphate buffer (pH 6.0) and 0.5 ml of filtrate to give a final reaction volume of 2.2 ml. The oxidation of the substrate (ABTS) was monitored by the increase in the absorbance at 420 nm using Shimadzu UV-1800 spectrophotometer (ELICO, India) over 90 s at 30 °C, using ε = 3.6 × 104 cm^−1^ M^−1^. Enzymatic activity was expressed as 1U = 1 μmol of ABTS oxidized per min at 25 °C (Vinothkumar et al. 2011).

## Results and discussion

The effect of process parameters (independent variables) on the decolourization and degradation of textile dye wastewater was studied. The second-order polynomial coefficients for each term of the equation Eqs. (3), (4) were determined using the Design-Expert 7.1.5. The experimental and predicted values of percentage decolourization and degradation are given in Table 2.

3 $$ \begin{gathered} {\text{\% Decolourization}}=69.7 8 - 0 . 1 3A - 1 . 5 4B + 2 . 3 0C + 14 . 5 6D + 1 . 6 8AB - 1 . 5 0AC - 2 . 1 3AD\; + \hfill \\ 2 . 2 8BC - 6 . 2 8BD - 0 . 0 7 5CD - 8 . 1 6A^ 2 - 4 . 1 0B^ 2 - 0 . 3 6C^ 2 - 6 . 9 5D^ 2 \hfill \\ \end{gathered} $$

4 $$ \begin{gathered} {\text{\% COD reduction}}=78.72 + 0 . 0 9 2A - 0 . 7 6B + 3 . 4 8C + 14 . 3 0D + \hfill \\ 0. 7 5AB - 2 . 0 0AC - 2 . 6 8AD\; + 2 . 5 5BC - 0 . 6 8BD - 1 . 4 0CD - 10 . 4 4 { }A^ {2 } - 1 . 9 1B^{ 2} - 2 . 5 5C^{ 2} - 4 . 1 0D^{{ 2 { }}} \hfill \\ \end{gathered} $$

where *A*, *B*, *C* and *D* are the coded values of the process variables, pH, temperature (^o^C), agitation speed (rpm) and wastewater concentration, respectively.

The results were analysed by using analysis of variance (ANOVA) and are given in Table 3. The ANOVA of the quadratic regression model indicates that the model is significant. In this work, the model *F* value 19.79 and 34.04 for decolourization and COD reduction implies that the models are significant. The smaller the magnitude of *P*, more significant is the corresponding coefficient. *P* value less than 0.05 indicates that the model terms are significant. From the *P* values, it was found that the variables *C*, *D*, *BD*, *A*^2^, *B*^2^ and *D*^2^ were significant model terms for decolourization and *C*, *D*, *A*^2^, *C*^2^ and *D*^2^ were significant model terms for COD reduction. From the ANOVA table, it was found that the linear effect of dye wastewater concentration is more significant for textile dye wastewater treatment followed by agitation speed. Also, the interactive effect of temperature and dye wastewater concentration is significant foe dye decolourization.

**Table 3:** ANOVA for the decolourization and COD reduction of textile dye wastewater

Source	% Decolourization			% COD reduction		
	Coefficient factor	*F*	*P* > *F*	Coefficient factor	*F*	*P* > *F*
Model	69.78	19.79	<0.0001	78.72	34.04	<0.0001
*A*	−0.13	0.017	0.8990	0.092	0.014	0.9074
*B*	−1.54	2.23	0.1572	−0.76	0.96	0.3440
*C*	2.30	4.97	0.0426	3.48	20.24	0.0005
*D*	14.56	199.24	<0.0001	14.30	341.09	<0.0001
*A* × *A*	−8.16	33.84	<0.0001	−10.44	98.26	<0.0001
*B* × *B*	−4.10	8.54	0.0112	−1.91	3.30	0.0906
*C* × *C*	−0.36	0.066	0.8008	−2.55	5.87	0.0295
*D* × *D*	−6.95	24.53	0.0002	−4.10	15.17	0.0016
*A* × *B*	1.68	0.88	0.3643	0.75	0.31	0.5848
*A* × *C*	−1.50	0.71	0.4152	−2.00	2.22	0.1581
*A* × *D*	−2.13	1.42	0.2540	−2.68	3.98	0.0659
*B* × *C*	2.28	1.62	0.2236	2.55	3.62	0.0780
*B* × *D*	−6.28	12.34	0.0034	−0.68	0.25	0.6226
*C* × *D*	−0.075	1.73E−003	0.9671	−1.40	1.09	0.3142
*R* ^2^	0.9519			0.9715		
Pred *R*^2^	0.9038			0.9429		
Adj *R*^2^	0.8240			0.8357		
Std. Dev.	3.5700			2.6800		
C.V	5.79 %			3.79 %		
Adeq precision	16.730			21.429		

The predicted *R*^2^ of 0.8240 (decolourization), 0.8357 (COD reduction) was in reasonable agreement with the adjusted *R*^2^ of 0.9038 (decolourization) and 0.9429 (COD reduction). The fit of the model is also expressed by the coefficient of regression *R*^2^, which is found to be 0.9519 for decolourization and 0.9715 for COD reduction, indicating that more than 95 % of the variability in the response could be explained by the model. This implies that the prediction of experimental data is quite satisfactory. Adequate precision measures the signal to noise ratio. A ratio greater than 4 is desirable. In this work, the ratio was found to be 16.730 and 21.429 for decolourization and COD reduction, which indicates an adequate signal.

The magnitude of coefficient factors in Table 3 gives the positive contribution of agitation speed and wastewater concentration and negative contribution of pH and temperature on dye wastewater decolourization. For COD reduction, pH, agitation speed and dye wastewater concentration show the positive contribution, whereas temperature has a negative effect.

**Fig. 1:**
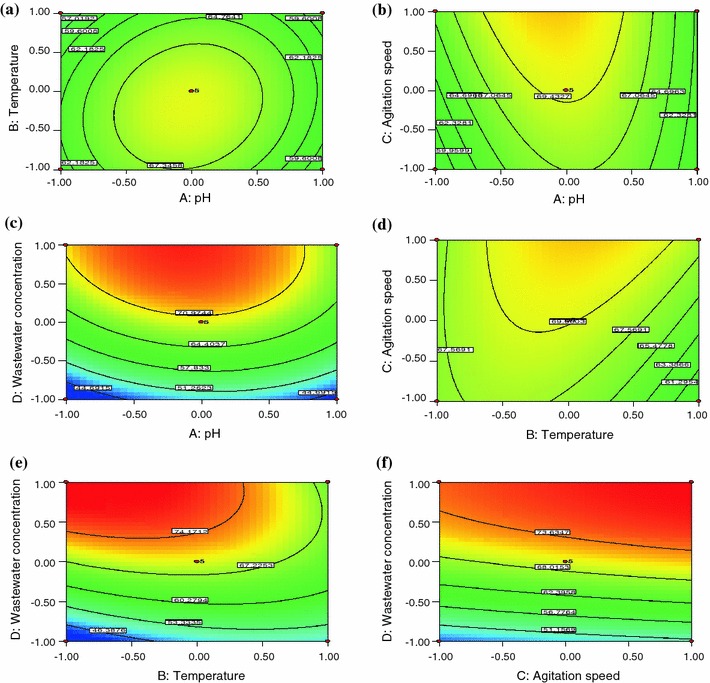
Contour plot showing the interactive effect of **a** pH and temperature, **b** pH and agitation speed, **c** pH and wastewater concentration, **d** temperature and agitation speed, **e** temperature and wastewater concentration, and **f** agitation speed and wastewater concentration on decolourization of textile dye wastewater

**Fig. 2:**
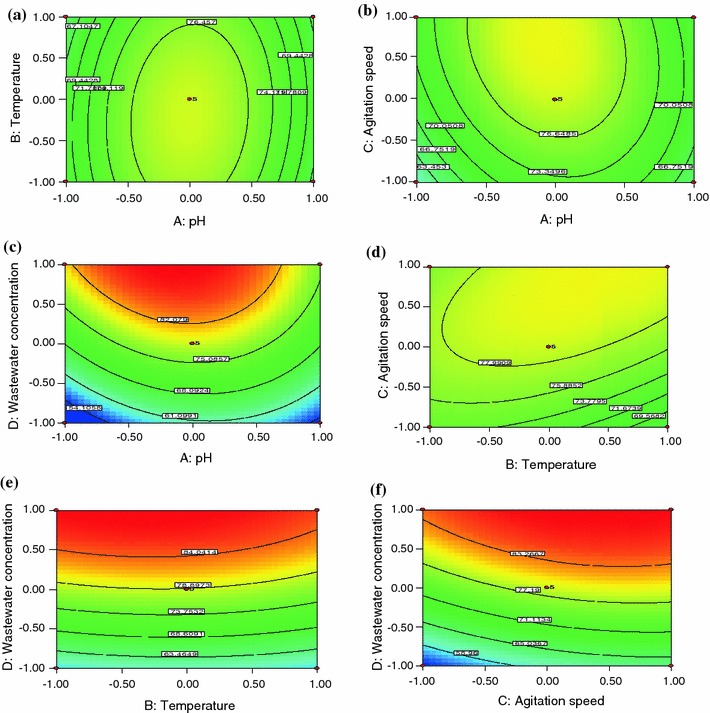
Contour plot showing the interactive effect of **a** pH and temperature, **b** pH and agitation speed, **c** pH and wastewater concentration, **d** temperature and agitation speed, **e** temperature and wastewater concentration and **f** agitation speed and wastewater concentration on COD reduction of textile dye wastewater

To investigate the interactive effect of two factors on the decolourization and degradation of dye wastewater, response surface methodology was used and contour plots were drawn. Response surface plots as a function of two factors at a time, maintaining all other factors at fixed levels, are more helpful in understanding both the main and the interactive effects of two factors. The response surface curves for the decolourization and degradation of textile dye wastewater are shown in Figs. 1 and 2. The nature of the response surface curves shows the interaction between the variables. The elliptical shape of the curve indicates good interaction between the two variables and circular shape indicates no interaction. From the figures, it is observed that the elliptical nature of the contour in graphs depicts the mutual interactions of all the variables. There is a relatively significant interaction between every two variables and a maximum predicted yield as indicated by the surface confined in the smallest ellipse in the contour diagrams.

### Decolourization of textile dye wastewater

Figure 1a shows the interactive effect of pH and temperature on textile dye decolourization. From the figure, it was inferred that increase in pH (up to 6.4) increases the dye decolourization efficiency. After that, the decolourization efficiency decreases. A similar trend was observed in Fig. 1b and c. The pH has a major effect on the efficiency of dye decolourization, and the optimal pH for colour removal is often between 6.0 and 7 for most of the dyes. The pH tolerance of decolourizing fungi is quite important for decolourization of textile dye wastewater.

It is clear from Fig. 1a that percentage decolourization of dye increased with an increase in temperature from 23 to 26.5 °C. The percentage decolourization of dye decreased with further increase in temperature up to 33 °C. The decolourizing activity was significantly suppressed at higher temperatures. This may be due to the loss of cell viability or deactivation of the enzymes responsible for decolourization. Hence the optimum temperature for maximum decolourization of textile dye wastewater using *Ganoderma lucidum* was 26.5 °C. From Fig. 1b, it was observed that the percentage of decolourization increased with increase in agitation speed up to 200 rpm. This shows that oxygen is needed for the microorganism to degrade the dye wastewater. A similar trend is observed in Fig. 1d, f. The decrease in initial textile dye wastewater concentration increases the decolourization. This is clearly depicted in Fig. 1c, e and f. From the figures, it is inferred that the percentage removal of dyes increases with decrease in initial dye concentration. This is because at higher concentrations, the chemicals and other pollutants present in the dye wastewater inhibit the growth of microorganism.

### Degradation of textile dye wastewater

pH is one of the important factors in the treatment of textile dye wastewater by microorganism. The interactive effect of pH and temperature on biodegradation of textile wastewater using *Ganoderma lucidum* is shown in Fig. 2a. From the figure, it was observed that increase in pH up to 6.6 increased the COD reduction. Further increase in pH leads to decrease in COD reduction. The interactive effects of other parameters are shown in Fig. 2b–f. The trend observed for other parameters was similar to the decolourization profile. The optimum conditions were also the same, except agitation speed (175), for COD reduction.

The second-order polynomial equation obtained from RSM was used to find the optimum conditions. The equation was solved in MATLAB 7.0. The optimum condition for the maximum decolourization was found to be: pH 6.6, temperature 26.5 °C, agitation speed 200 rpm and dye wastewater concentration 1:2.The optimal conditions predicted using RSM has been validated using experiments. At the optimized condition, the maximum colour removal and COD reduction were found to be 81.4 and 90.3 %, respectively.

**Fig. 3:**
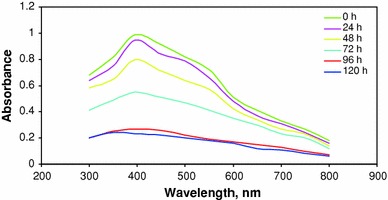
UV spectra of textile dye wastewater decolourization by *Ganoderma lucidum* at the optimum condition

At the optimum condition, decolourization of textile wastewater was studied by analysing the supernatants at different time intervals, in a UV-spectrophotometer in the range of 300–800 nm. The results obtained are shown in Fig. 3. A peak is observed at λ_max_ 395 nm in the UV–Vis spectra. The peak decreases as the day progresses, which shows the decolourization of textile dye using *Ganoderma lucidum* at the end of the 5th day of operation. The peak at 395 nm attains nearly zero, which shows almost complete decolourization of the textile dye.

**Fig. 4:**
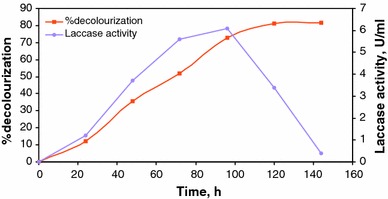
Laccase activity during the decolourization of textile dye wastewater by *Ganoderma lucidum*

The laccase activity was measured at these optimum conditions and is shown in Fig. 4. From the figure, it was inferred that the laccase activity increased till the 4th day and reached a maximum of 6.1 U/ml; thereafter, the laccase enzyme activity decreases. The decolourization process increases with increase in enzyme production and decreases with decrease in enzyme activity. After 6 days, no enzyme activity was found and the decolourization process stopped. This clearly shows that the decolourization process occurs by the action of the laccase enzyme.

## Kinetics

In this study, first-order model, diffusional model and Singh model were tried to fit the experimental data obtained from the batch degradation of textile dye wastewater using *Ganoderma lucidum*.

### First-order model

The First-order model is

5 $$ - \frac{dC_{s}}{dt} = k_{1}C_S $$

On integration between known limits and rearranging, the above model becomes

6 $$ \ln \left( {\frac{{C_{\text{so}} }}{{C_{s} }}} \right) = k_{1} t $$

where *C*_so_ is the initial substrate concentration in mg COD/L, *C*_*s*_ is the substrate concentration in mg COD/L, *t* is the degradation time in h, *k*_1_ is the first-order rate constant in h^−1^.

### Diffusional model

The diffusional model is given by

7 $$ - \frac{dC_s}{dt} = k_{2} C_{s}^{0.5} $$

when integrated between the known limits, the above equation becomes

8 $$ \sqrt {C_{s} } - \sqrt {C_{\rm{so}} } = \frac{{k_{2} }}{2}t $$

where *k*_2_ = rate constant for diffusional model.

### Singh model

The Singh model is given by

9 $$ - \frac{dC_{s}}{dt} = \frac{{k_{3} C_{s} }}{1 + t} $$

Integrating the above equation between the proper limits, it becomes

10 $$ \ln \left( {\frac{C_s}{{C_{\text{so}}}}} \right) = - k_{3} \ln (1 + t) $$

where *k*_3_ is the rate constant for the Singh model.

**Fig. 5:**
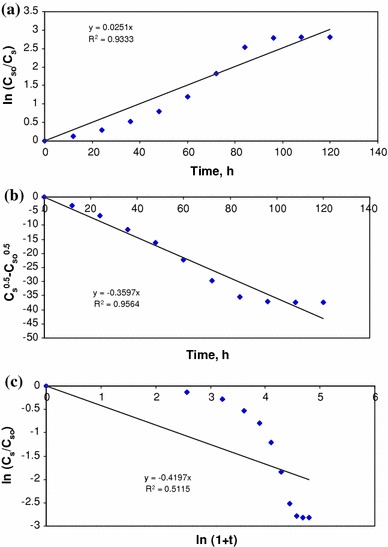
Kinetic plots for degradation of textile dye wastewater. **a** First-order model, **b** diffusional model and **c** Singh model

The data obtained from the batch study were fitted to the first-order, diffusional and Singh model and are shown in Figs. 5a–c. The rate constants, *k*_1,_*k*_2_ and *k*_3_ were calculated from the slope of the straight line by the least square (LSQ) fit in the figures. The values of *k*_1,_*k*_2_ and *k*_3_ were 0.0251, −0.3597 and 0.4197, respectively. The determination coefficient (*R*^2^) for first-order, diffusional and Singh model was 0.9333, 0.9383 and 0.5115, respectively. The high *R*^2^ value for the first order indicates the fitness of the model for the degradation of textile dye wastewater. The negative value of the rate constant for diffusional model and low *R*^2^ value for Singh models show the inability of these models to describe this process.

## Conclusions

In this study, a white rot fungi, *Ganoderma lucidum*, was utilized to treat the textile dye wastewater. RSM was applied to optimize the process parameters. From the results, it was found that a maximum of 81.4 % colour removal and 91.3 % COD reduction occurs at the optimized condition. The UV spectrum confirms the decolourization. Various models were tried to study the kinetics of textile dye degradation. From the results, it was found that the degradation of textile dye wastewater follows first-order kinetics. Hence, it was concluded that *Ganoderma lucidum* could be utilized for the effective treatment of textile dye wastewater.
